# Multi-omics analysis identifies *Sphingomonas* and specific metabolites as key biomarkers in elderly Chinese patients with coronary heart disease

**DOI:** 10.3389/fmicb.2025.1452136

**Published:** 2025-04-23

**Authors:** Xiaoshan Zhou, Tianlong Zhang, Sixiang Jia, Shudong Xia

**Affiliations:** ^1^Department of Critical Care Medicine, The Fourth Affiliated Hospital of School of Medicine, and International School of Medicine, International Institutes of Medicine, Zhejiang University, Yiwu, China; ^2^Department of Cardiology, The Fourth Affiliated Hospital of School of Medicine, and International School of Medicine, International Institutes of Medicine, Zhejiang University, Yiwu, China

**Keywords:** coronary heart disease, gut microbiota, metabolites, metabolomics, 16s rRNA gene sequencing

## Abstract

**Background:**

Abnormal component changes of gut microbiota are related to the pathogenesis and progression of coronary heart disease (CHD), and gut microbiota-derived metabolites are key factors in host-microbiome interactions. This study aimed to explore the key gut microbiota and metabolites, as well as their relationships in CHD.

**Methods:**

Feces samples and blood samples were collected from CHD patients and healthy controls. Then, the obtained feces samples were sent for 16s rRNA gene sequencing, and the blood samples were submitted for metabolomics analysis. Finally, conjoint analysis of 16s rRNA gene sequencing and metabolomics data was performed.

**Results:**

After sequencing, there were no significant differences in Chao 1, observed species, Simpson, Shannon, Pielou’s evenness and Faith’s PD between the CHD patients and controls. At phylum level, the dominant phyla were *Firmicutes*, *Bacteroidetes*, *Proteobacteria*, and *Actinobacteria*. At genus level, the abundance of *Sphingomonas*, *Prevotella*, *Streptococcus*, *Desulfovibrio*, and *Shigella* was relatively higher in CHD patients; whereas *Roseburia*, *Corprococcus*, and *Bifidobacterium* was relatively lower. Randomforest analysis showed that *Sphingomonas* was more important for CHD. Through metabolomic analysis, a total of 155 differential metabolites were identified, and were enriched in many signaling pathways. Additionally, the AUC of the conjoint analysis (0.908) was higher than that of gut microbiota species (0.742).

**Conclusion:**

In CHD patients, the intestinal flora was disordered, as well as *Sphingomonas* and the identified differential metabolites may serve as was candidate biomarkers for CHD occurrence and progression.

## 1 Introduction

With the rapid growth of China’s economy and the improvement of people’s living standards, coronary heart disease (CHD) has increasingly become a global health problem and a major cause of morbidity and premature death worldwide (Albarrati et al., 2018). It is estimated that more than 11 million people in China suffer from CHD, and the number is expected to grow steadily in the coming decades (Moran et al., 2010; Dorje et al., 2019). CHD is characterized by the formation of arterial plaques composed primarily of lipids, calcium and inflammatory cells that narrow the coronary lumen, leading to attack or persistent angina (Li et al., 2018). The rupture of these plaques can contribute to the formation of thrombosis, thus leading to myocardial infarction and death (Tian et al., 2019). It has been reported that the risk factors of CHD are varied, including hypertension, unhealthy diet, diabetes, hypercholesterolemia, smoking, excessive drinking and depression (Yang et al., 2019; Amann et al., 2021). Nowadays, surgical treatment (percutaneous coronary intervention and coronary artery bypass grafting) and drug therapy (aspirin, low molecular heparin, urokinase, and statins) are used to manage CHD, and drug therapy is the basis of all treatments (Hsue and Waters, 2019). However, the timing of surgical treatment is important and technically difficult; and the long-term use of drugs can cause some adverse effects (Liang and Wang, 2021). Therefore, it is necessary to further explore the pathogenesis of CHD, so as to unearth underlying biomarkers or therapeutic targets for CHD.

Gut microbiota is a general term for the microorganisms existing in the human gut, which is composed of 1 × 10^14^ communities and more than 1,000 species of bacteria (Cheng et al., 2021). Gut microbiota, influenced by diet, genetic, host and environmental factors, plays important roles in regulating metabolism, immunity and the nervous system, maintaining a delicate balance with the host (Liu et al., 2020). For healthy people, there is a dynamic balance between harmful and beneficial bacteria in gut microbiota to maintain the healthy state of the host (Jin et al., 2019). However, imbalance of gut microbiota can lead to structural disorder of the bacteria, and disrupt basic metabolic processes of the host, resulting in the occurrence and development of some diseases (Trøseid et al., 2020). More and more evidence has suggested that gut microbiota is implicated in the occurrence and progression of various cardiometabolic diseases, such as obesity, heart failure, cardiovascular disease (CVD), diabetes, dyslipidemia and hypertension (Jie et al., 2017; Kazemian et al., 2020; Wu and Chiou, 2021). A previous study of Han et al. (2021) showed that in the acute myocardial infarction (AMI) patients, the abundance of phylum *Firmicutes*, genera *Pseudobutyrivibrio*, and L*achnospiraceae ND3007 group* was lower, while the abundance of phylum *Bacteroidetes* and genera *Desulfovibrio*, *Butyricimonas*, and *Acidaminococcus* was higher compared to the healthy controls, which indicated the dysbiosis of gut microbiota in AMI. Another study demonstrated that statins could modulate the gut microbiota of acute coronary syndrome (ACS) patients to a healthier state, i.e., decreasing pathogenic bacteria, such as *Paracenobacteria Merdae*, while elevating beneficial bacteria, like *Anaerostipes hadrus* and *Bifidobacterium longum subsp. longum* (Hu et al., 2021). These reports indicated that gut microbiota may participate in the development of multiple cardiometabolic diseases. However, changes of gut microbiota composition in CHD remain unclear and need to be further investigated.

In addition, gut microbiota, considered as “bioreactors,” can ferment food and break them into functional metabolites or microbial products through regulating many metabolic processes in the host, including energy homeostasis, glucose metabolism, and lipid metabolism (Macfarlane and Macfarlane, 2003; Wu and Chiou, 2021). Cui et al. (2018) employed metabolomics to analyze the blood samples of chronic heart failure (CHF) patients, and found that in CHF patients, beneficial metabolites like orotic acid were decreased; and harmful metabolites such as sphingosine 1-phosphate were increased. Trimethylamine N-oxide (TMAO), one of the metabolites formed by gut microbiota, has been reported to be positively correlated with CVD, and increase the size of plaques, triggering prethrombotic platelet function and promoting the growth of arterial thrombosis (Randrianarisoa et al., 2016; Yang et al., 2019). Metabolomics analysis enables rapid discovery of active metabolites that alter cellular physiology, thereby, these metabolites can be used as biomarkers for disease diagnosis and prediction (Rinschen et al., 2019). Teruya et al. (2021) applied metabolomics to find that 33 metabolites including quinolinic acid, ergothioneine, glycerophosphocholine, and amino acid may be biomarkers of dementia patients. However, the metabolic profiles of CHD patients and potential metabolites involved in CHD are still lacking.

Therefore, this study collected feces samples and blood samples from CHD patients and healthy controls, and then 16s rDNA sequencing and metabolomics analysis were used to investigate the key gut microbiota and metabolites closely associated with CHD. Our research will provide direct evidence for gut microbiota dysbiosis of CHD and provide new insights for treatment of CHD.

## 2 Materials and methods

### 2.1 Patient recruitment and sample collection

From March 2021 to July 2021, 20 CHD in-hospital patients (disease group, verified by coronary angiography), and 20 control individuals (normal group) were recruited from The Fourth Affiliated Hospital, Zhejiang University School of Medicine (ZJU4H). The diagnosis of CHD was established based on the World Health Organization (WHO) criteria and confirmed through coronary angiography. As defined by the WHO, CHD is a condition marked by the narrowing or obstruction of coronary arteries caused by the accumulation of atherosclerotic plaques (Pega et al., 2021). This reduction in arterial diameter leads to decreased blood flow to the heart muscle, potentially resulting in myocardial infarction, angina, or other forms of ischemic heart disease. The criteria for the controls were coronary stenosis of <25% as assessed by invasive coronary angiograms or coronary CT angiography. The exclusion criteria consisted of subjects that: (i) received antacids, probiotics, antibiotics, or antimicrobial agents within 30 days before sample collection, (ii) had an organic disease of the digestive system, diabetes, or hypertension, and (iii) had gastrointestinal surgery.

And the fresh fecal (2–5 g) and blood (5 mL) samples were obtained from each subject under the hospital diet. Then the fecal samples were transferred into sterile collecting pipes and frozen at −80°C immediately. The blood samples were centrifuged and the upper serum was collected and stored at −80°C. The basic clinical information of the enrolled subjects is displayed in Table 1 and Supplementary Table 1.

**Table 1:** The basic clinical information of coronary heart disease (CHD) patients and control individuals.

Index	Group			
	CHD (*N* = 20)	Control (*N* = 20)	Total (*N* = 40)	*P*-value
Age				
Mean (SD)	61.2 (12.4)	55.2 (13.1)	58.2 (13.0)	0.339
Median [Min, Max]	63.0 [36.0, 79.0]	57.0 [31.0, 72.0]	59.0 [31.0, 79.0]	
Sex				
Man	14 (70.0%)	13 (65.0%)	27 (67.5%)	0.945
Woman	6 (30.0%)	7 (35.0%)	13 (32.5%)	
Systolic_pressure				
Mean (SD)	133 (19.6)	130 (16.8)	132 (18.1)	0.836
Median [Min, Max]	136 [92.0, 166]	132 [102, 161]	132 [92.0, 166]	
Diastolic_pressure				
Mean (SD)	77.3 (9.43)	80.6 (10.1)	78.9 (9.80)	0.528
Median [Min, Max]	76.5 [57.0, 94.0]	78.5 [58.0, 100]	77.5 [57.0, 100]	
BMI				
Mean (SD)	25.4 (3.40)	23.4 (2.84)	24.4 (3.25)	0.235
Median [Min, Max]	25.7 [20.6, 33.2]	23.7 [18.7, 27.8]	24.3 [18.7, 33.2]	
Triglyceride				
Mean (SD)	1.30 (0.378)	1.65 (1.10)	1.47 (0.822)	0.824
Median [Min, Max]	1.35 [0.620, 2.07]	1.28 [0.670, 5.53]	1.34 [0.620, 5.53]	
Missing	0 (0%)	1 (5.0%)	1 (2.5%)	
Heart_rate				
Mean (SD)	72.0 (11.7)	79.7 (11.7)	75.9 (12.2)	0.0509
Median [Min, Max]	67.5 [54.0, 95.0]	80.5 [46.0, 100]	77.5 [46.0, 100]	
Total cholesterol				
Mean (SD)	3.41 (0.809)	4.38 (1.15)	3.88 (1.09)	0.0232
Median [Min, Max]	3.33 [2.25, 5.46]	4.24 [2.69, 6.96]	3.62 [2.25, 6.96]	
Missing	0 (0%)	1 (5.0%)	1 (2.5%)	
Low density lipoprotein				
Mean (SD)	1.96 (0.700)	2.55 (0.781)	2.25 (0.788)	0.0702
Median [Min, Max]	1.92 [0.860, 3.56]	2.48 [1.56, 4.19]	2.13 [0.860, 4.19]	
Missing	0 (0%)	1 (5.0%)	1 (2.5%)	
High density lipoprotein				
Mean (SD)	0.977 (0.235)	1.12 (0.215)	1.05 (0.234)	0.231
Median [Min, Max]	1.04 [0.510, 1.30]	1.15 [0.780, 1.52]	1.12 [0.510, 1.52]	
Missing	0 (0%)	1 (5.0%)	1 (2.5%)	
Weight				
Mean (SD)	68.2 (11.8)	63.6 (11.8)	65.9 (11.9)	0.48
Median [Min, Max]	68.8 [51.0, 92.5]	64.0 [46.0, 90.0]	65.8 [46.0, 92.5]	
Smoke				
No	12 (60.0%)	17 (85.0%)	29 (72.5%)	0.209
Yes	8 (40.0%)	3 (15.0%)	11 (27.5%)	
Aspirin				
No	2 (10.0%)	8 (40.0%)	10 (25.0%)	0.0907
Yes	18 (90.0%)	12 (60.0%)	30 (75.0%)	
Clopidogrel				
No	14 (70.0%)	19 (95.0%)	33 (82.5%)	0.115
Yes	6 (30.0%)	1 (5.0%)	7 (17.5%)	
Statins				
No	0 (0%)	1 (5.0%)	1 (2.5%)	0.599
Yes	20 (100%)	19 (95.0%)	39 (37.5%)	

### 2.2 16s rRNA gene sequencing of fecal samples and bioinformatic analysis

The collected fecal samples from the all subjects were submitted to Yanzai Biotechnology (Shanghai) Co., Ltd. (Shanghai, China) for 16s rRNA gene sequencing based on the Illumina MiSeq platform. Total DNA was extracted from the fecal samples using a Fecal DNA Extraction Kit (Takara Biomedical Technology Co., Ltd., Beijing, China), and the quality and concentration of the extracted total DNA were assessed using 1.2% agarose gel electrophoresis and Nanodrop. After that, a primer set (341F ACTCCTACGGGAGGCAGCA/806R CGGACTACHVGGGTWTCTAAT) of V3-V4 region was used to amplify the target fragment, and then the amplified products were purified. The sequencing library was constructed using the TruSeq Nano DNA LT Library Prep kit (Illumina) following the manufacturer’s instructions. After quality testing using a Agilent High Sensitivity DNA Kit on Agilent Bioanalyzer, the DNA samples were sequenced on the MisSeq sequenator.

QIIME 2 (2019.4^1^) was used for gut microbiota bioinformatics analysis. The raw sequencing data were filtered, denoised, merged and chimera removed using DADA2, and amplicon sequence variants (ASVs) were obtained. Then, ASVs were mapped to Greengenes database (Release 13.8^2^) in QIIME 2 to assign operational taxonomic units (OTUs) according to the criteria of 98% sequencing similarity. Thereafter, the diversity of gut microbiota, different species composition at phylum and genus levels between the disease and normal groups, as well as underlying pathways involved in CHD were further analyzed.

### 2.3 Isolation of metabolites and metabolomics analysis of blood serum

The obtained blood serum from the all participants were used for metabolomic analysis. The blood serum samples (100 μL) were transferred to a new 2 mL centrifuge tube, and 400 μL pre-cooled methanol was added. After vortex oscillated for 60 s, the samples were centrifuged at 12,000 rpm for 10 min at 4°C, and the supernatant was transferred to a new tube. After concentrated to dry in vacuum, the dried powder was redissolved in 150 μL 80% methanol solution (80%) with 2-chlorobenzalanine (4 ppm), and then filtered through a 0.22 μm membrane. The obtained samples were used for liquid chromatography-mass spectrometry (LC–MS) detection.

A Thermo Ultimate 3000 system equipped with an ACQUITY UPLC^®^ HSS T3 column (1.8 μm, 2.1 × 150 mm, Waters), and a mass spectrometer (Orbitrap 120, thermo) were employed for LC–MS. The temperature of sample injector and column was, respectively, 8°C and 40°C. The flow rate was 0.25 mL/min, and the injection volume was 2 μL. The mobile phases for positive were 0.1% formic acid in water (C) and 0.1% formic acid in acetonitrile (D); and for negative were 5 mM ammonium formate water (A) and acetonitrile (B). The elution program was set as follows: 2% B/D, 0–1 min; 2–50% B/D, 1–9 min; 50–98%, 9–12 min; 98% B/D, 12–13.5 min; 98–2% B/D, 13.5–14 min; 2% D, 14–20 min (positive)/ 2% B, 12–17 min (negative). The spray voltage of MS for positive and negative modes was, respectively, 3.5 kV and 2.5 kV; sheath gas and auxiliary gas were set at 30 and 10 arbitrary units; and the capillary temperature was 325°C. Full scanning was carried out with a resolution of 60,000, and the scanning range of 100–1,000 m/z.

The raw data generated by LC–MS were converted to a mzXML format (xcms input file format) using Proteowizard software (v3.0.8789), and then the CXMS software of R package (v3.3.2) was applied for peaks identification, peaks filtration, peaks alignment with the parameters of bw = 5, ppm = 15, peakwideth = c (530), mzwid = 0.015, mzdiff = 0.01 and method = “centWave.” A data matrix consisted of mass to charge ration (m/z), retention time and peak intensity was obtained. After normalization, the metabolites were annotated, and differential metabolites were screened based on the thresholds of *p*-value ≤ 0.05 and VIP ≥ 1. Receiver operating characteristic (ROC) and area under curve (AUC) were used to evaluate the sensitivity and specificity of the identified differential metabolites (Lin et al., 2014).

### 2.4 Functional analysis

The functional potential of the gut microbiota was predicted using **PICRUSt2** (Phylogenetic Investigation of Communities by Reconstruction of Unobserved States). The **Amplicon Sequence Variants (ASVs)** obtained from 16S rRNA gene sequencing were used as input. These ASVs were mapped to the **Greengenes database** (version 13.8) for taxonomic annotation. PICRUSt2 employs a **phylogenetic placement approach** to infer the functional content of microbial communities based on the known functional annotations of closely related reference genomes. The predicted functional content was then mapped to the **Kyoto Encyclopedia of Genes and Genomes (KEGG) pathway** and **MetaCyc pathway** databases to identify and annotate metabolic pathways. Statistical analysis was performed to compare pathway abundances between groups, and pathways with an adjusted *p*-value < 0.05 were considered significant. The results were visualized using heatmaps and bar plots to highlight differences in functional pathways between CHD patients and healthy controls. Finally, the differential metabolites identified through metabolomics analysis were submitted for **KEGG pathway enrichment analysis** to further explore their potential roles in metabolic processes related to CHD.

### 2.5 Conjoint analysis of 16s rRNA gene sequencing and metabolomics data

Pearson correlation analysis was used to calculate the correlation coefficient and *p* value of the two groups; and then redundancy analysis was used to analyze the relationships between gut microbiota at genus level and differential metabolites, and *p* < 0.05 was considered as the significant level.

## 3 Results

### 3.1 Alpha biodiversity of gut microbiota in CHD

**Figure 1:**
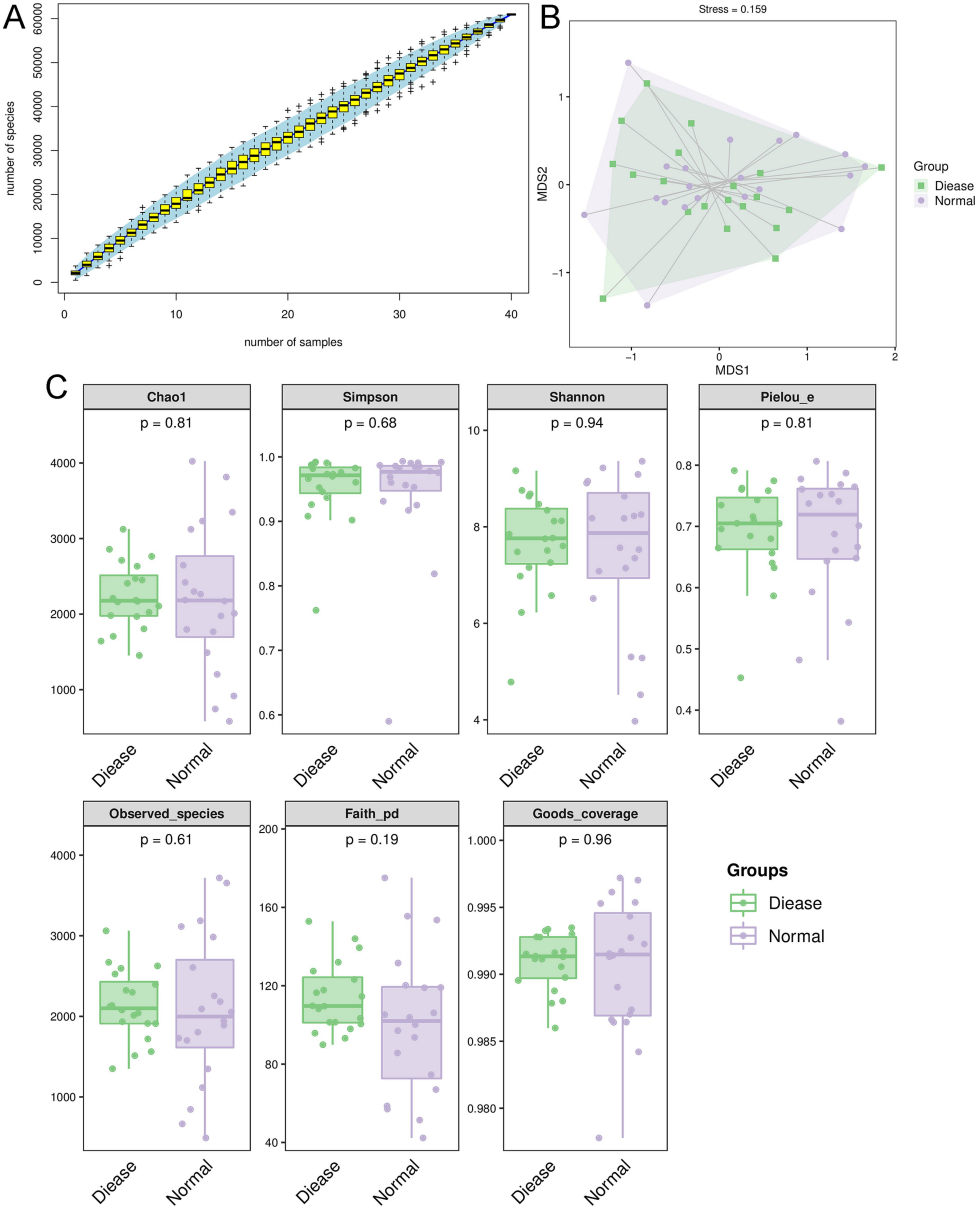
Overall diversity of gut microbiota in coronary heart disease (CHD). **(A)** Species accumulation curves analysis. **(B)** Nonmetric multidimensional scale analysis (NMDS) showed the value of stress was 0.159 (<0.2). **(C)** The alpha diversity analyses based on Chao 1, observed species, Simpson, Shannon, Pielou’s evenness and Faith’s PD indexes.

In order to understand the roles of gut microbiota in CHD, the feces of CHD patients and control individuals were sent for 16s rRNA gene sequencing. It is clear that in the current sequencing, the species accumulation curve tended to be stable with the increase of sample size (Figure 1A), indicating that the sequencing depth and sample size were sufficient to reflect the species composition of gut microbiota and capture most of the diversity. Nonmetric multidimensional scale analysis (NMDS) showed there was no obvious clustering of microbial composition in the CHD and control subjects; as well as the value of stress was 0.159 (<0.2, Figure 1B), which suggested that the result of NMDS was reliable. The Good’s coverage in the CHD and control groups were, respectively, 0.9909 ± 0.002 and 0.9606 ± 0.005 (Figure 1C), implying that the present sequencing contained basically most species, and can be used for further analyses.

Thereafter, the indexes of Chao 1, observed species, Simpson, Shannon, Pielou’s evenness and Faith’s PD were calculated. It is obvious that there were no significant differences in values of Chao 1, observed species, Simpson, Shannon, Pielou’s evenness and Faith’s PD between the CHD patients and control individuals (*p* > 0.05, Figure 1C). Taken together, it can be inferred that CHD did not alter the alpha biodiversity of gut microbiota compared to the control participants.

### 3.2 The compositions of specific gut microbiota in CHD

**Figure 2:**
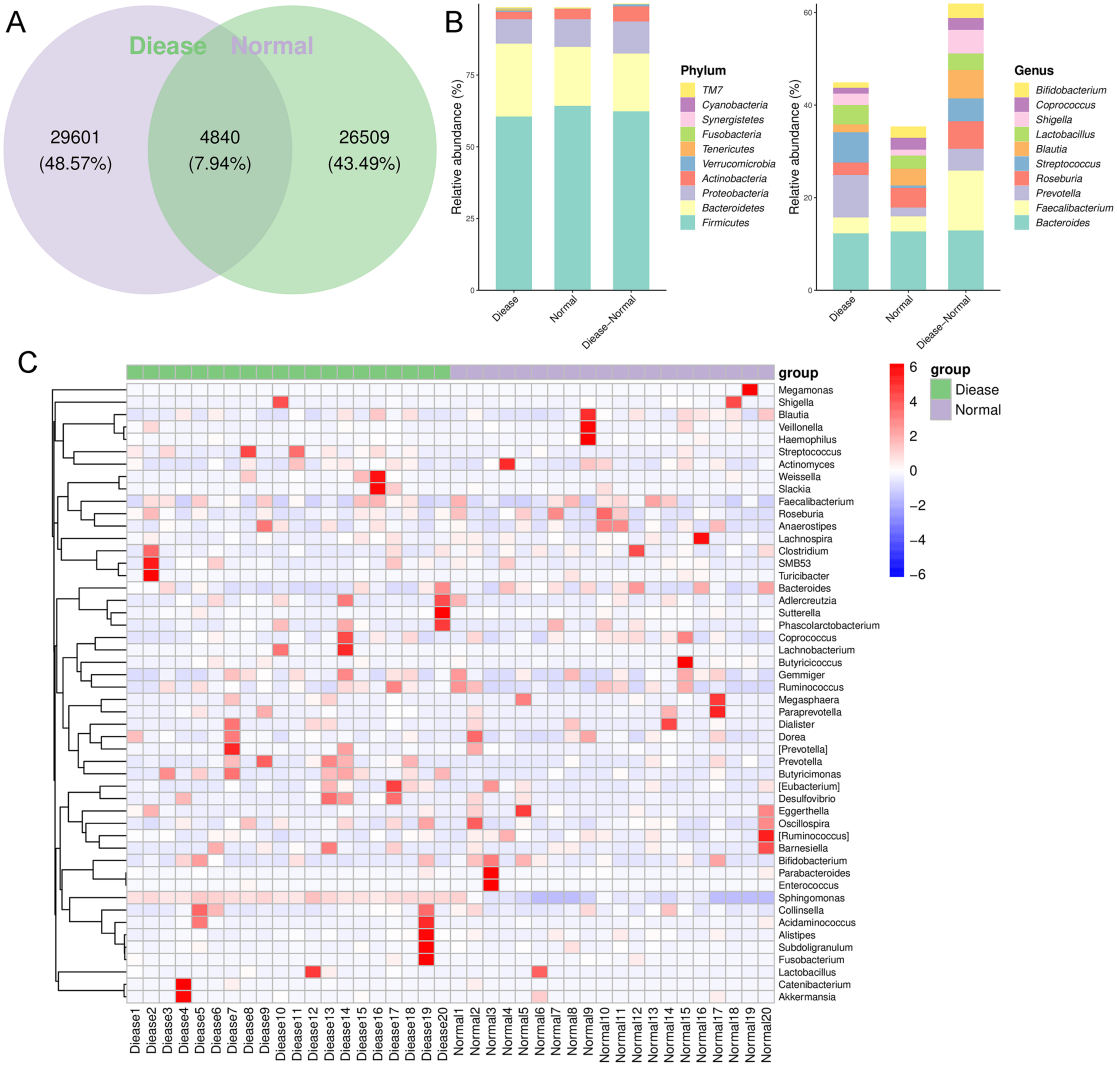
The gut microbial composition in CHD. **(A)** Venn diagram of operational taxonomic unit (OUT) in the CDH patients and control individuals. **(B)** The relative abundance of top 10 phyla and genera in the CDH patients and control subjects. **(C)** The clustering heatmap of the prominent OTUs (top50) assigned to genus level among each sample.

After that, we further explored changes in the compositions of specific gut microbiota at phylum and genus levels. As shown in Figure 2A, it was found that there were 34,441 OTUs and 31,349 OTUs in the disease group and normal group, respectively, which included 4,840 shared OTUs. From the level of phylum, the dominant phyla were *Firmicutes*, *Bacteroidetes*, *Proteobacteria*, *Actinobacteria*, and *Verrucomicrobia* (Figure 2B). However, from the aspect of genus, we found that dominant genera were *Bacteroides*, *Faecalibacterium*, *Prvotella*, *Roseburia*, and *Streptococcus* (Figure 2B). Additionally, the relative abundance of *Prevotella*, *Streptococcus*, *Lactobacillus*, and *Shigella* was higher in the CHD patients than that in the control participants; whereas the abundance of *Roseburia*, *Blautia*, *Corprococcus*, and *Bifidobacterium* was relatively higher in the control subjects compared to the CHD patients (Figure 2B).

**Figure 3:**
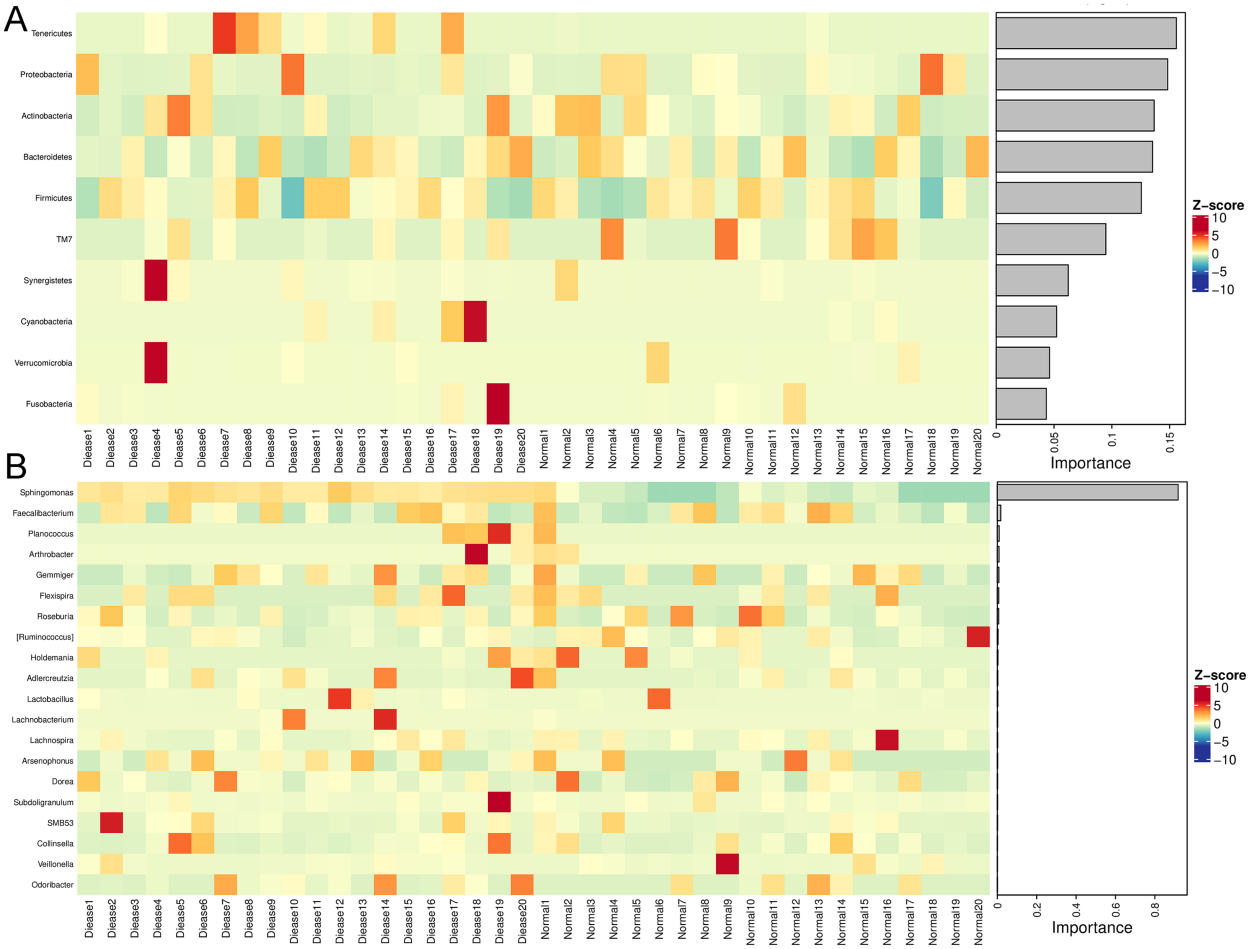
Randomforest analysis of gut microbiota in CHD. **(A)** The randomforest diagram of gut microbiota at phylum level. **(B)** The randomforest diagram of gut microbiota at genus level.

Following, a clustering heatmap analysis was carried out on the top50 genera between the two groups (Figure 2C). It can be seen that *Sphingomonas*, *Slackia*, *Lachnobacterium*, *Prevotella*, *Butyrcimonas*, and *Desulfovibrio* were relatively enriched in the CHD patients compared with the control individuals. Then, randomforest analysis showed that *Tenericutes*, *Proteobacteria*, *Acitinobacteria*, and *Bacteroidetes* were important phyla for CHD (Figure 3A); and the key genus for CHD was *Sphingomonas* (Figure 3B).

### 3.3 Functional analyses of the annotated gut microbiota

**Figure 4:**
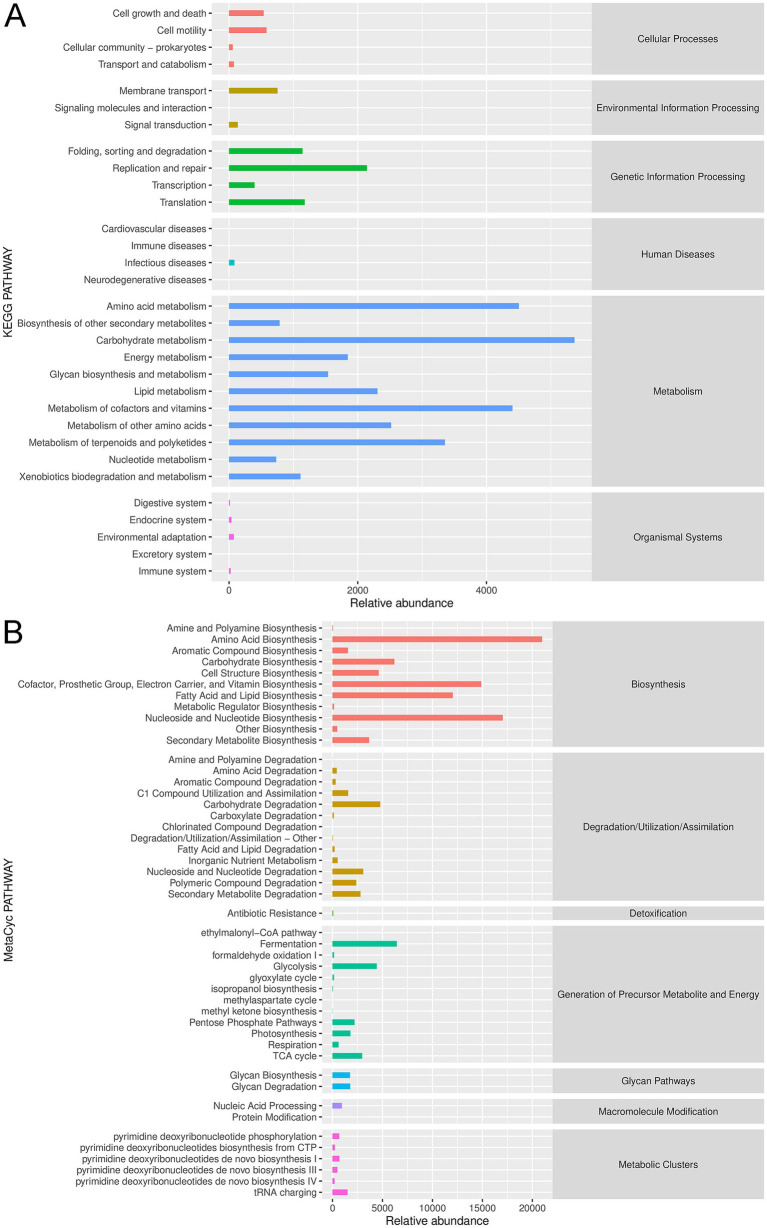
Functional analysis of the annotated gut microbiota. **(A)** Kyoto Encyclopedia of Genes and Genomes (KEGG) pathway enrichment of the annotated gut microbiota. **(B)** MetaCyc pathway analysis of the annotated gut microbiota.

The annotated gut microbiota was submitted for functional analysis, including KEGG pathways and MetaCyc pathways analyses. KEGG pathway enrichment demonstrated that the annotated gut microbiota was enriched in “carbohydrate metabolism,” “amino acid metabolism,” “metabolism of cofactors and vitamins,” “metabolism of terpenoids and polyketides” and “lipid metabolism” in the metabolism term; and “replication and repair,” “translation” and “folding, sorting and degradation” in the genetic information processing term; and “cell growth and death” and “cell motility” in the cellular processes term; as well as “membrane transport” and “signaling transduction” in the environmental information processing (Figure 4A). Furthermore, MetaCyc results showed that gut microbiota could play important role in CHD through “amino acid biosynthesis,” “nucleoside and nucleotide biosynthesis/degradation,” “cofactor, prosthetic group, electron carrier, and vitamin biosynthesis,” “secondary metabolism biosynthesis/degradation,” “carbohydrate degradation,” “fermentation,” “glycolysis,” “TCA cycle,” “pentose phosphate pathways,” “glycan biosynthesis/ degradation,” “tRNA charging,” and “pyrimidine deoxyribonucleotide phosphorylation” (Figure 4B).

### 3.4 Identification of differential metabolites in CHD

**Figure 5:**
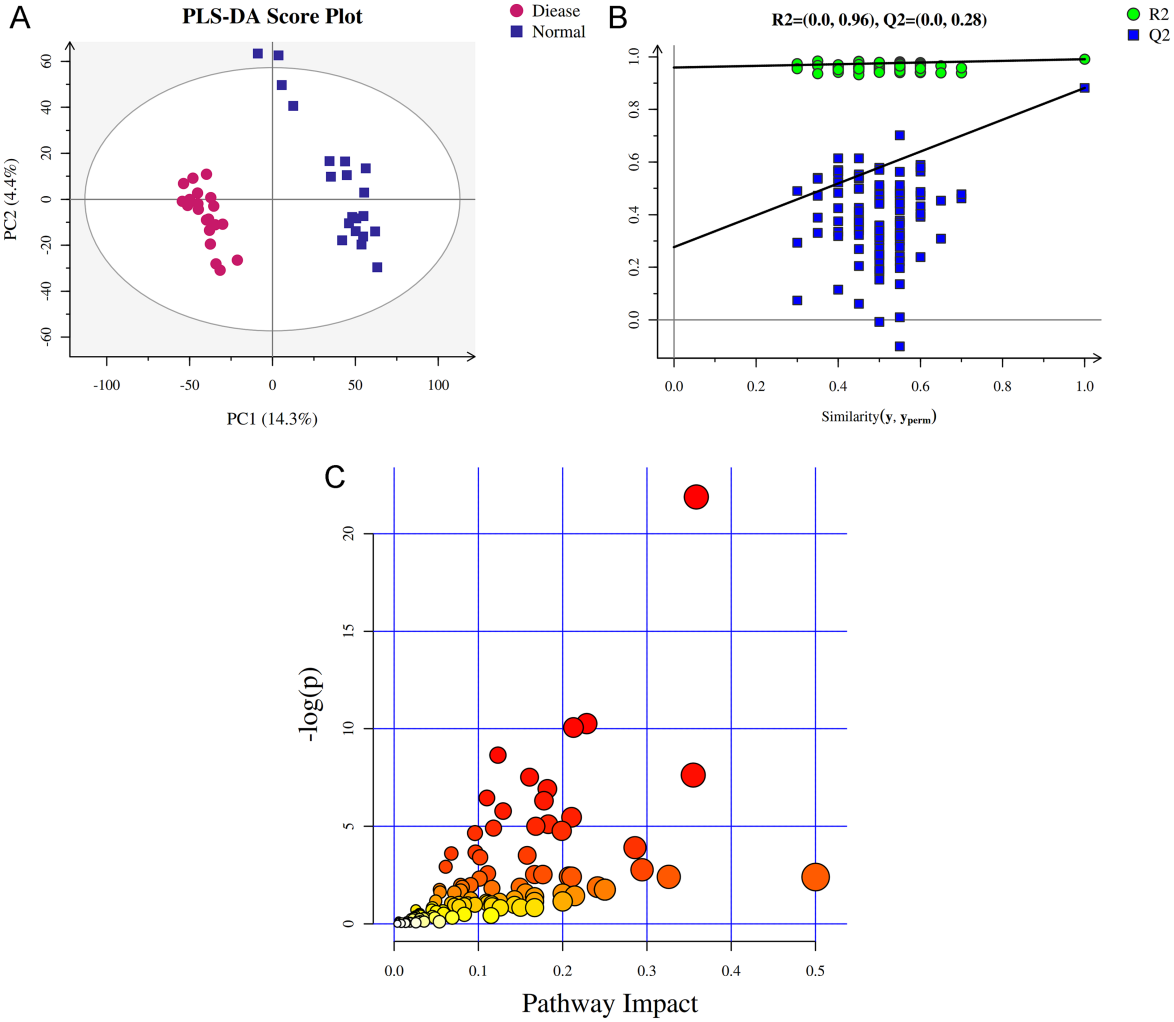
The blood serum metabolomic profile in CHD. **(A)** The score diagram of Partial Least Squares-Discriminant Analysis (PLS-DA) based on blood serum samples. **(B)** Displacement test diagram of PLS-DA. **(C)** KEGG pathways map of the identified differential metabolites.

We further performed the metabolomics analysis of bold serum for CHD patients and control participants. As shown in Supplementary Figure 1, quality control (QC) samples were densely distributed and good reproducible, which indicated the system was stable and the data was reliable. In QC samples, the proportion of characteristic peaks with relative standard deviation (RSD) < 30% WAS 84% (Supplementary Figure 1), illustrating that the metabolomics data were good and reliable, and conducive to the detection of biomarkers. Additionally, Partial Least Squares-Discriminant Analysis (PLS-DA) model showed the samples in the disease group were significantly distinguished from the samples in the normal group (Figure 5A), as well as the values of R2 and Q2 in this model were, respectively, 0.991 and 0.882 (Figure 5B), which indicated the evaluation model was effective and reliable, and could be used for subsequent secondary structure analysis.

According to the thresholds of VIP ≥ 1 and *p*-value ≤ 0.05, a total of 155 differential metabolites were screened, including 99 upregulated ones and 56 downregulated ones (Table 2). The clustering heatmap of the identified differential metabolites were displayed in Supplementary Figure 2 using agglomerate hierarchical clustering method. Compared with the control participants, the relative concentrations of metabolites phenacetin, 1-methyladenosine, 9,10-DHOME, dodecanedioic acid, GMP, N6-acetyl-L-lysine, gitogenin and creatine were significantly elevated in the CHD patients; while the relative concentrations of acetylcholine chloride, o-phosphoethanolamine, quinolinic acid, trehalose, tobramycin and diaminopimelic acid were reduced in the CHD participants (Table 2).

**Table 2:** All differential metabolites between control individuals and coronary heart disease patients.

	VIP	Fold change	log2(FC)	*p* value	FDR
Phenacetin	1.18553	15.617	3.965	4.54E-07	5.04E-05
Dodecanedioic acid	1.908746	11.572	3.5326	4.54E-07	4.92E-05
Creatine	1.701693	6.5058	2.7017	1.81E-05	0.000326
Gitogenin	1.258032	6.0301	2.5922	0.000104	0.001072
Phenmetrazine	2.316949	4.1988	2.07	2.36E-06	0.000104
Oxoglutaric acid	1.812159	4.1779	2.0628	7.58E-06	0.000201
4,5-Dihydroorotic acid	2.204628	3.9884	1.9958	3.42E-07	4.79E-05
N-[(3a,5b,7a)-3-hydroxy-24-oxo-7-(sulfooxy)cholan-24-yl]-Glycine	1.112346	3.6963	1.8861	0.000758	0.004647
Maleic acid	1.887571	3.3165	1.7297	4.54E-06	0.000136
4-Pyridoxic acid	1.371738	3.2686	1.7087	0.000921	0.005253
2-O-(alpha-D-Mannosyl)-D-glycerate	1.660888	3.2471	1.6992	2.92E-05	0.000449
Isocitric acid	1.681158	3.2168	1.6856	5.25E-05	0.000668
3,4-Dihydroxyhydrocinnamic acid	1.30448	3.1588	1.6594	0.003057	0.013417
Glycochenodeoxycholic acid	1.059019	3.1348	1.6484	0.010581	0.034557
Biliverdin	1.065255	3.118	1.6406	0.000921	0.005382
Catechol	2.02395	2.9321	1.5519	8.6E-06	0.000219
4-Hydroxycinnamic acid	1.481582	2.9051	1.5386	0.0002	0.001709
Phenylacetylglutamine	1.373208	2.7898	1.4802	0.001349	0.007022
L-Phenylalanine	2.227579	2.6977	1.4317	1.2E-06	7.03E-05
3-Methylindole	1.082333	2.6759	1.42	0.00556	0.021142
Bilirubin	1.400503	2.5557	1.3537	0.000758	0.004647
16-Hydroxypalmitate	1.693513	2.5196	1.3332	4.68E-05	0.000611
L-2-Amino-3-oxobutanoic acid	1.066464	2.3689	1.2442	0.001481	0.007718
N-[(2S)-2-Amino-2-carboxyethyl]-L-glutamate	1.705818	2.3676	1.2434	5.25E-05	0.000668
GMP	2.317182	2.3413	1.2273	2.96E-07	4.42E-05
2-Propylmalate	1.95265	2.255	1.1731	1.41E-05	0.000272
Acetylphosphate	1.549707	2.2428	1.1653	0.000509	0.003342
3-Hydroxyphenylacetic acid	1.38116	2.231	1.1577	0.002799	0.012578
Vitexin	1.089289	2.2144	1.1469	0.007113	0.025507
4-Guanidinobutanoic acid	1.713666	2.2045	1.1404	1.41E-05	0.000295
Ribitol	1.194625	2.1234	1.0864	2.6E-05	0.000415
Pyrrolidonecarboxylic acid	1.278558	2.1219	1.0854	0.004703	0.018393
3-Methylthiopropionic acid	1.818538	2.0312	1.0224	4.54E-06	0.000136
Dhurrin	1.436371	2.0283	1.0203	0.007712	0.027103
9(S)-HPODE	1.512025	2.0178	1.0128	0.000179	0.001584
Pantothenic acid	1.486845	1.9885	0.99165	8.29E-05	0.000908
9,10-DHOME	1.77927	1.9873	0.99079	1.2E-06	7.03E-05
Xanthine	1.662858	1.9659	0.97521	1.41E-05	0.000272
Lidocaine	1.878093	1.9504	0.96375	0.000275	0.002135
Phenylpropanolamine	1.988867	1.9238	0.94395	0.000222	0.001837
4-Hydroxyphenylacetaldehyde	2.079808	1.923	0.94335	1.58E-06	8.48E-05
4-Quinolinecarboxylic acid	1.4427	1.9028	0.92815	0.003639	0.015321
N-Acetyl-D-tryptophan	1.244305	1.8899	0.91833	0.002799	0.012247
1-Methyladenosine	2.105591	1.8845	0.91415	4.54E-06	0.000151
D-Glucose	1.406796	1.8556	0.89191	0.001349	0.007184
1,3-Dihydro-(2H)-indol-2-one	1.353302	1.8521	0.88917	0.003336	0.014059
Phenylethylamine	1.166687	1.8517	0.88886	0.015479	0.04658
Creatinine	1.04659	1.78	0.83188	0.003336	0.014348
O-Succinyl-L-homoserine	2.080243	1.7772	0.82957	3.99E-06	0.000128
2-Isopropylmalic acid	1.720999	1.7373	0.79688	0.000144	0.001354
L-Asparagine	1.007519	1.7301	0.79083	0.014364	0.044592
Aminoadipic acid	1.90314	1.714	0.77737	2.04E-05	0.000368
N6-Acetyl-L-lysine	2.230201	1.6964	0.76252	3.94E-07	5.01E-05
N-Formyl-L-methionine	1.977055	1.692	0.75872	3.29E-05	0.000484
Erythritol	1.644237	1.6883	0.75555	0.000375	0.002752
3-Hydroxymethylglutaric acid	1.316197	1.6523	0.72449	0.002561	0.011728
Rimantadine	1.042011	1.639	0.71279	0.020735	0.059535
L-Isoleucine	1.322898	1.5787	0.65873	0.002341	0.010926
N,N-Dimethylhistidine	1.395155	1.5779	0.65805	0.002799	0.012247
Anhalamine	1.233867	1.5697	0.65053	0.001349	0.007022
Prunasin	1.24128	1.5217	0.60566	0.008355	0.028772
FMN	1.23167	1.5145	0.59884	0.002799	0.012578
Uric acid	1.206473	1.503	0.58782	0.002341	0.010926
Undecanoic acid	1.250193	1.5016	0.58652	0.004703	0.018393
Glycerophosphocholine	1.127094	1.4758	0.56147	0.006557	0.023858
Epsilon-caprolactam	1.907618	1.4436	0.52963	0.00046	0.003092
Adipic acid	1.450385	1.4164	0.50227	0.000836	0.005013
N-Alpha-acetyllysine	1.282101	1.4142	0.49999	0.003639	0.015321
cis-Aconitate	1.242097	1.4043	0.48988	0.003639	0.015321
Uridine	1.122918	1.3848	0.46966	0.007113	0.025421
Citramalic acid	1.327291	1.3757	0.4602	0.000758	0.004647
Benzamide	1.581982	1.3735	0.45781	0.000247	0.001979
L-Leucine	1.46586	1.3583	0.4418	0.001014	0.005782
N-methyl-L-glutamic Acid	1.095815	1.3577	0.44115	0.010581	0.034557
Vanylglycol	1.268893	1.3551	0.43836	0.004703	0.018393
Neocembrene	1.870779	1.3539	0.43716	6.61E-05	0.000777
5-Methylthioadenosine	1.531213	1.3528	0.43591	0.000179	0.00158
1H-Indole-3-carboxaldehyde	1.249154	1.3268	0.40797	0.00432	0.017501
2,6-Dimethoxyphenol	1.52917	1.3224	0.40313	0.000687	0.004175
2-Furoate	1.185449	1.2859	0.36282	0.003639	0.015321
Gamma-Glutamylalanine	1.267519	1.2722	0.34732	0.012345	0.039536
Pyridoxamine	1.221648	1.2564	0.32925	0.007712	0.027189
Glucosamine	1.05601	1.2515	0.3237	0.020735	0.059535
L-Valine	1.790042	1.2494	0.32128	5.25E-05	0.000668
L-Tyrosine	1.157048	1.2458	0.31709	0.008355	0.028772
L-Cystine	1.432023	1.2356	0.30525	0.001481	0.007509
Mesaconate	1.059796	1.2311	0.29996	0.020735	0.058191
Thyrotropin releasing hormone	1.074954	1.2298	0.29844	0.000338	0.002492
Capric acid	1.758715	1.2287	0.29708	0.000116	0.001149
7-Methylguanine	1.396456	1.2281	0.29641	0.002799	0.012247
Sebacic acid	1.051473	1.2262	0.29419	0.041124	0.100435
Oxalacetic acid	1.992975	1.2011	0.26433	3.07E-06	0.00011
4-Nitrophenol	1.144062	1.1893	0.25013	0.023903	0.064795
Choline	1.425813	1.1721	0.22911	0.001116	0.006082
Myo-Inositol	1.146689	1.142	0.19157	0.023903	0.064795
Pelargonic acid	1.061161	1.1344	0.18196	0.041124	0.097686
Gamma-Glutamylcysteine	1.30365	1.1094	0.14976	0.005115	0.019861
Methyl beta-D-galactoside	1.642616	1.0286	0.040746	0.000247	0.002013
Imidazol-5-yl-pyruvate	1.314027	1.0261	0.037141	0.011433	0.037216
Alpha-Santonin	1.344555	0.94315	−0.08444	0.003057	0.013092
Isophorone	1.022448	0.93454	−0.09768	0.04388	0.105169
Diaminopimelic acid	1.536219	0.92935	−0.1057	0.000836	0.004877
1,1-Dimethylbiguanide	1.014765	0.84635	−0.24067	0.020735	0.059535
13(S)-HPOT	1.276588	0.83948	−0.25242	0.002341	0.010926
Sphingosine	1.461377	0.83655	−0.25747	0.002341	0.010635
Ascorbate	1.281539	0.82602	−0.27575	0.007712	0.027189
Saccharopine	1.277684	0.82275	−0.28147	0.00604	0.022323
Threonic acid	1.034718	0.8188	−0.28842	0.027483	0.072043
Erucic acid	1.542439	0.7929	−0.33479	0.000416	0.002975
2-Heptanone	1.526107	0.78896	−0.34197	0.000144	0.001357
Dihydrouracil	1.050395	0.77236	−0.37266	0.019292	0.056279
3-Dehydroshikimate	1.686888	0.76177	−0.39257	0.000161	0.001468
Deoxycholic acid	1.842048	0.75828	−0.39919	4.17E-05	0.000575
Citrulline	1.485544	0.75124	−0.41266	0.001782	0.008618
Phenylacetic acid	1.061202	0.7506	−0.41389	0.020735	0.059535
Folic acid	1.937893	0.74092	−0.43261	7.58E-06	0.000185
Gluconic acid	1.426563	0.73953	−0.43533	0.003057	0.013417
Indoleglycerol phosphate	1.580061	0.73455	−0.44507	0.000921	0.005253
Spermidine	1.138233	0.72404	−0.46586	0.04388	0.102401
Trehalose	1.165864	0.71248	−0.48907	0.001782	0.008911
Fructose 1,6-bisphosphate	1.077604	0.71101	−0.49206	0.038515	0.09302
L-Histidine	1.526892	0.70292	−0.50857	0.000161	0.001463
Triacetate lactone	1.273865	0.70069	−0.51316	0.00432	0.017232
5-Guanidino-3-methyl-2-oxopentanoate	1.114318	0.70056	−0.51341	0.023903	0.066822
(2R,3R)-3-Methylglutamyl-5-semialdehyde-N6-lysine	1.081811	0.69377	−0.52747	0.002139	0.009925
Dehydroepiandrosterone	1.1536	0.66215	−0.59478	0.02227	0.063069
N2-gamma-Glutamylglutamine	1.603426	0.65151	−0.61813	9.28E-05	0.000984
L-Glutamine	1.349041	0.64419	−0.63444	0.012345	0.038989
Acetylcholine chloride	2.478521	0.6207	−0.68802	1.43E-07	4.14E-05
3-Methyl-L-tyrosine	1.785866	0.61356	−0.70472	4.68E-05	0.000622
L-Threonine	1.819506	0.59664	−0.74508	0.000116	0.001155
Alpha-D-Glucose	1.394597	0.58245	−0.77979	0.000622	0.003995
3-(2-Hydroxyphenyl)propanoic acid	1.88794	0.55471	−0.8502	1.81E-05	0.000338
Citric acid	1.599549	0.52239	−0.93681	7.41E-05	0.000844
Dehydroepiandrosterone sulfate	1.121374	0.52019	−0.94289	0.029441	0.075824
8-Hydroxyquinoline	1.411668	0.5065	−0.98138	0.0002	0.001711
Tobramycin	1.993714	0.49796	−1.0059	1.1E-05	0.000253
Glycocholic acid	1.116974	0.48421	−1.0463	0.00604	0.022323
Hydroquinone	1.481197	0.47122	−1.0855	0.002139	0.009925
2-Dehydro-3-deoxy-D-xylonate	1.614671	0.45962	−1.1215	0.000305	0.002347
Nicotine	1.286395	0.44974	−1.1528	0.002561	0.011728
Bovinocidin	1.615586	0.42589	−1.2315	0.000247	0.001979
Taurine	1.337972	0.37403	−1.4188	2.6E-05	0.000425
p-Hydroxyphenylacetic acid	1.831916	0.37051	−1.4324	8.29E-05	0.000914
L-Aspartate-semialdehyde	1.753674	0.35686	−1.4866	8.29E-05	0.000908
Gentamicin X2	1.392728	0.34701	−1.527	0.001481	0.007509
4-Acetamidobutanoic acid	1.522384	0.33819	−1.5641	9.28E-05	0.000984
2-Ketobutyric acid	1.272717	0.32723	−1.6116	0.006557	0.023858
O-Toluidine	1.717093	0.32346	−1.6283	0.000144	0.001357
L-4-Hydroxyphenylglycine	1.298582	0.30497	−1.7132	0.003966	0.016093
Phosphoglycolic acid	1.63914	0.30328	−1.7213	5.9E-05	0.000719
Palmitic acid	1.551572	0.26971	−1.8905	0.001116	0.006082
9,12,13-TriHOME	1.171146	0.25824	−1.9532	0.033718	0.084368
Quinolinic acid	1.250689	0.23942	−2.0624	0.000836	0.004877
O-Phosphoethanolamine	1.404009	0.090082	−3.4726	2.06E-06	9.6E-05

Next, ROC curves of these identified differential metabolites were drawn, and AUC was calculated. ROC curves were used to evaluate and screen the potential biomarkers, and AUC was employed to assess the sensitivity and specificity of biomarkers for predicting event occurrence. It is clear that the AUC values of 1-methyladenosine, 9,10-DHOME, acetylcholine chloride, dodecanedioic acid, GMP, and N6-acetyl-L-lysine were 0.925, 0.96, 0.991, 0.975, 0.98, and 0.979, respectively (Supplementary Figure 3). The results implied that these identified differential metabolites, including 1-methyladenosine, 9,10-DHOME, acetylcholine chloride, dodecanedioic acid, GMP, and N6-acetyl-L-lysine, could be used as the biomarkers to predict the occurrence and development of CHD.

### 3.5 KEGG pathway analysis of the identified differential metabolites

KEGG is a database of systematic analysis of gene function and genomic information. The identified differential metabolites were applied for KEGG pathway enrichment analysis. Based on the results of Figure 5C and Supplementary Table 2, we found that the differential metabolites were significantly enriched in the pathways of “mTOR signaling pathway,” “central carbon metabolism in cancer,” “valine, leucine and isoleucine biosynthesis,” “insulin signaling pathway,” “glucagon signaling pathway,” “sphingolipid signaling pathway,” “FoxO signaling pathway,” “Citrate cycle (TCA cycle),” “AMPK signaling pathway,” “HIF-1 signaling pathway” and “cGMP-PKG signaling pathway.”

### 3.6 Conjoint analysis of 16s rRNA gene sequencing and metabolomics

**Figure 6:**
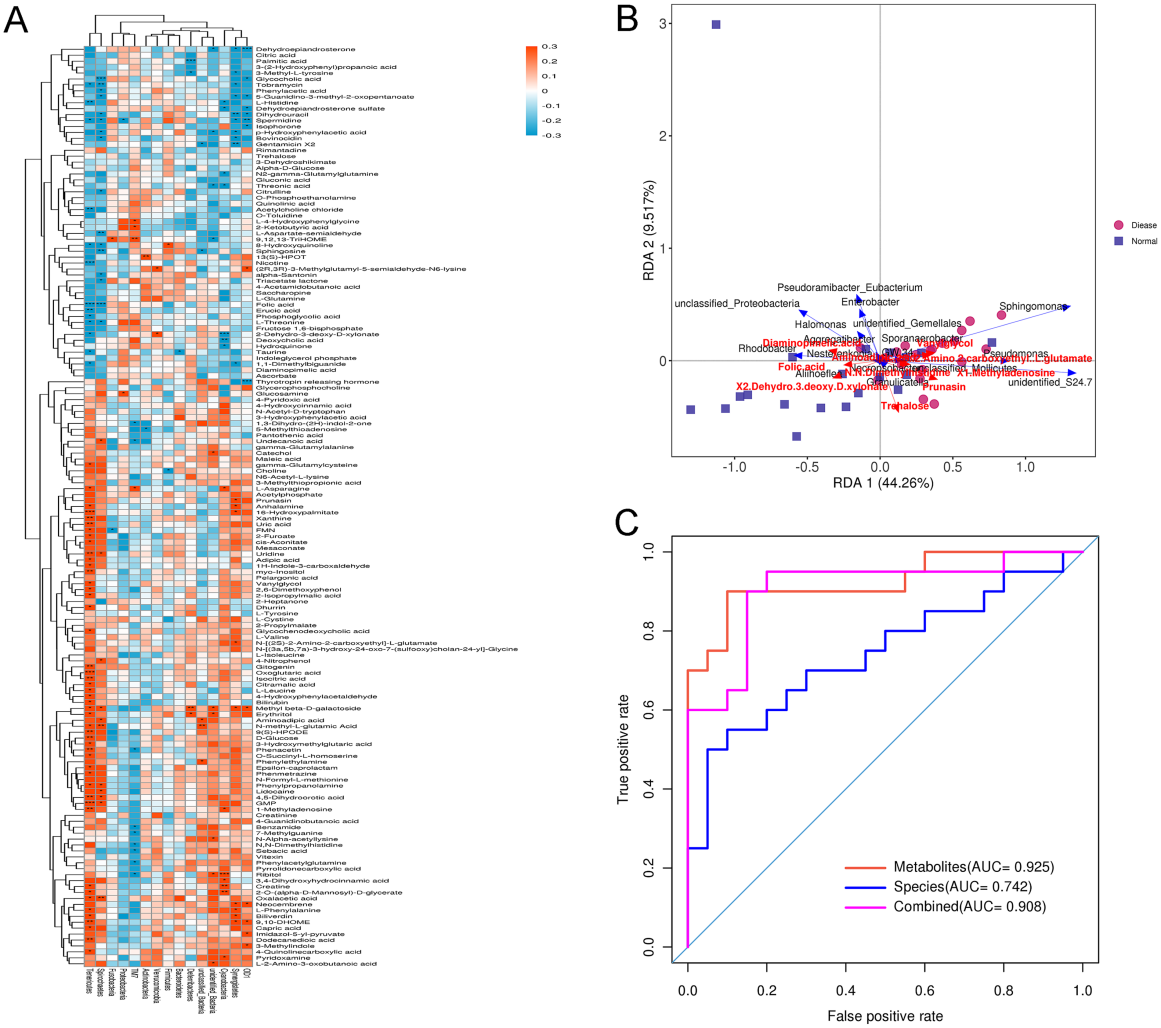
Conjoint analysis of 16s rRNA gene sequencing and metabolomics. **(A)** The associated heatmap between the gut microbiota at phylum level and the differential metabolites. Different colors and depth represent the size of the correlation coefficient. Red represents a positive correlation, while blue represents a negative correlation. *0.01 < *p* < 0.05; **0.0001 < *p* < 0.01; ***p < 0.0001. **(B)** Redundancy analysis (RDA) of gut microbiota at genus level corresponding to differential metabolite. The acute angle between two variables represents a positive correlation, that is, a synergistic effect. The obtuse Angle between two variables means a negative correlation, that is, an antagonistic effect. **(C)** Receiver operating characteristic (ROC) and area under curve (AUC) of the gut microbiota at genus level, differential metabolites and conjoint analysis.

Further to explore the relationships between gut microbiota and differential metabolites, the conjoint analysis of 16s rRNA gene sequencing and metabolomics data were performed. Pearson correlation coefficient analysis showed the relationship between gut microbiota at phylum level and the differential metabolites (Figure 6A). At the phylum level, *Firmicutes* were significantly negatively correlated with choline, while evidently positively correlated with 8-hydroxyquinoline. *Verrucomicrobiota* were markedly positively correlated with 2-dehydro-3-deoxy-D-xylonate and (2R,3R)-3-methylglutamyl-5-semialdehyde-N6-lysine. For *Proteobacteria*, it was evidently negatively related to spermidine, whereas significantly positively associated with glucosamine (Figure 6A). Besides, *Tenericutes* was found to be correlated with most differential metabolites (Figure 6A). After that, RAD and ROC were used to analyze the relationship between differential metabolites and gut microbiota at the genus level. For example, *Sphingomonae* had synergetic effects with vanylglycol, N-[(2S)-2-Amino-2-carboxyethyl]-L-glutamate, and 1-Methyladenosine; while had antagonistic effects with 2-dehydro-3-deoxy-D-xylonate and aminoadipic acid (Figure 6B). Additionally, *Enterobacter* had antagonistic effects with trehalose, but *Pseudomonas* had synergetic effects with vanylglycol. For the metabolite of N,N-Dimethylhistidine, five bacteria had antagonistic effects, including *Nesterenkonia*, *Aliihoeflea*, *Granulicatella*, *Necropsobacter*, and *Aggregatibacter*. Finally, the AUC values of differential metabolites, gut microbiota species and conjoint analysis were 0.925, 0.742, and 0.908 (Figure 6C). These indicated that the predictive effect of conjoint analysis was better than that of gut microbiota species alone, whereas slightly lower than that of differential metabolites alone.

## 4 Discussion

CHD is a common heart disease, which seriously affects the quality of life and mental state of patients. It was reported that the incidence of depressive symptoms in CHD patients was 77.23%(Im et al., 2018). The abnormal component changes of gut microbiota are related to the pathogenesis and progression of CHD. Gut microbiota-derived metabolites are key factors in host-microbiome interactions, and represent the potential biomarkers for early diagnosis, and show promising therapeutic targets in metabolic disorders (Agus et al., 2021). In this study, changes of gut microbiota and metabolites in CHD were analyzed using 16s rRNA gene sequencing and metabolomics. From the overall structure of the gut microbiota, we found that no significant difference in alpha biodiversity of gut microbiota between the CHD patients and control ones. A study of Hu et al. (2021) showed that the alpha biodiversity and overall structure of gut microbiota in ACS patients did not alter significantly compared with the healthy controls. Another study reported that the richness level (Chao) in the CHD was significantly higher than that in the health controls; whereas there were no significant differences in the diversity levels (Shannon and Simpson) (Wan et al., 2021). Taken together, we speculate that CHD may not influence the overall diversity and structure of gut microbiota, but this conclusion requires to be verified in larger sample size.

The compositions of specific gut microbiota were then analyzed at phylum and genus levels. It was found that *Firmicutes*, *Bacteroidetes*, *Proteobacteria*, *Actinobacteria*, and *Verrucomicrobia* were dominant phyla in CHD. At genus level, the relative abundance of *Sphingomonas*, *Prevotella*, *Streptococcus*, *Desulfovibrio*, and *Shigella* was relatively higher in CHD patients; whereas the abundance of *Roseburia*, *Corprococcus*, and *Bifidobacterium* was relatively lower. Randomforest analysis showed that *Sphingomonas* was more important for CHD. *Sphingomonas* is an aerobic, non-fermentative, opportunistic Gram-negative bacterium that is a rare cause of human infection, found mainly in patients with chronic diseases (e.g., diabetes), malignant tumors, and other immune deficiencies (Tang et al., 2022). *Sphingomonas* is a low-virulence bacterium, but if not diagnosed and treated early, it may lead to many complications, such as tricuspid valve endocarditis with pulmonary infarction (Tang et al., 2022), bacteremia with pyogenic spondylodiscitis (Dsouza et al., 2021), and splenic abscess (Birlutiu et al., 2022). A study of Higuchi et al. (2021) reported that the increased abundance of *Sphingomonas* could be associated with impaired immunity in the tumor microenvironment, which indicated that *Sphingomonas* may be a specific microbiota related to cancer progression, and may be used as a biomarker for thymoma in clinical. These findings, together with our results, it can be inferred that *Sphingomonas* may be closely related to CHD, and may serve as a crucial biomarker of intestinal flora for CHD development. However, the mechanisms of *Sphingomonas* in CHD need to be further investigated.

CHD is closely associated with inflammation, lipid compositions and metabolic disturbance. *Prevotella*, commonly identified in the fecal samples of CHD patients (Chen et al., 2021), primarily activates toll-like receptor 2 and exhibits increased inflammatory properties that promote the recruitment of mucosal Th17 immune response and neutrophil (Larsen, 2017). *Prevotella*-mediated mucosal inflammation can result in systemic transmission of inflammatory mediators, bacteria, and bacterial products, which in turn may contribute to the progression of systemic diseases, for example, rheumatoid arthritis, periodontitis, and bacterial vaginosis (Larsen, 2017). *Desulfovibrio* has been identified as an endotoxin-producing bacterium, and is higher in the intestinal tract of a CHD rat model (Peng et al., 2020). *Streptococcus* and *Shigella* are both harmful bacteria. The abundance of *Streptococcus* was found to be increased in atherosclerotic cardiovascular disease, which deviated from a healthy state (Jie et al., 2017). *Bifidobacterium*, a kind of probiotics, can improve the glucose tolerance of patients with hyperglycemia and other metabolic disorders via regulating the health of human intestinal flora (Ming et al., 2021). *Roseburia* and *Corprococcus* belong to butyrate-producing probiotics, are negatively correlated with the development of atherosclerotic lesions in genetically diverse mouse populations (Kasahara et al., 2018). Therefore, we speculated that the intestinal flora may be disordered in CHD patients, characterized by a decrease in beneficial bacteria (*Roseburia*, *Corprococcus*, and *Bifidobacterium*) and an increase in harmful pathogens (*Sphingomonas*, *Prevotella*, *Streptococcus*, *Desulfovibrio*, and *Shigella*).

It has been reported that metabolites in blood are associated with gut microbiome under various physiological and pathological conditions (Visconti et al., 2019). In our study, a total of 155 differential metabolites, including 99 upregulated and 56 downregulated ones, were identified. The AUC values of 1-methyladenosine, 9,10-DHOME, acetylcholine chloride, dodecanedioic acid, GMP, and N6-acetyl-L-lysine were all above 0.9, which indicated the identified differential metabolites could be used as biomarkers for CHD.

Among these metabolites, some have been supported by existing literature as being closely related to the development and progression of coronary heart disease (CHD). For example, 1-methyladenosine, a nucleoside metabolite, may be associated with the dysregulation of apoptosis and autophagy, both of which play important roles in the pathophysiology of CHD (Dong et al., 2019). Similarly, 9,10-DHOME, an oxidized lipid metabolite, may reflect enhanced oxidative stress and inflammatory responses in CHD patients, processes that are closely linked to the progression of atherosclerosis (Yang et al., 2019). Additionally, the decreased levels of acetylcholine chloride may be related to endothelial dysfunction, a key pathological feature of CHD (Penna et al., 2017).

In our study, we also identified some novel metabolites, such as dodecanedioic acid and N6-acetyl-L-lysine, which were significantly elevated in CHD patients but have not been explicitly reported in the literature in relation to CHD. These metabolites may represent novel findings from our research, but their specific mechanisms of action require further investigation. We believe that future studies should validate the diagnostic value of these metabolites in larger cohorts and explore their potential roles in the pathogenesis of CHD. Furthermore, we recognize that metabolite levels may be influenced by various factors, including disease severity, medication use, and other potential confounding variables. For instance, CHD patients are often treated with statins, antiplatelet drugs, and other medications, which may affect metabolite levels by modulating metabolic pathways (Hu et al., 2021). Therefore, we suggest that future studies consider stratified analyses of these factors to more accurately assess the relationship between metabolites and CHD. Additionally, we note that variations in disease severity may lead to fluctuations in metabolite levels, as the metabolic profiles of mild and severe CHD patients may differ. This is an area that we believe requires further research to elucidate.

Functional analysis showed these differential metabolites were enriched in many pathways, such as mTOR signaling pathway, sphingolipid signaling pathway, FoxO signaling pathway, TCA cycle, AMPK signaling pathway, HIF-1 signaling pathway and cGMP-PKG signaling pathway. Apoptosis and autophagy play essential roles in the occurrence, development and prognosis of CHD. Increased apoptosis and autophagy have been reported in patients with CHD (Dong et al., 2019). A previous study demonstrated that AMPK signaling pathway, FoxO signaling pathway, and mTOR signaling pathway participate in cell autophagy, thus playing a dual role in CHD (Dong et al., 2019). HIF-1 signaling is considered to be a key pathway of viral infection in the cardiovascular system, and has found to be involved in the formation and rupture of atherosclerotic plaques (Zhang et al., 2021). Besides, cGMP is a second messenger widely used in the nervous system, and PKG is one of the main effectors of cGMP, which can catalyze the phosphorylation of a variety of proteins, including ion channels. Penna et al. (2017) showed that obestatin could mediate cardiovascular function and promote cardiac protection through cGMP-PKG signaling pathway. TCA cycle is the central pathway of oxidative phosphorylation in cells and meets the requirements of bioenergy, biosynthesis and redox equilibrium (Anderson et al., 2018). Sphingolipid 1-phosphosphingol is a signaling lipid, and its production and signaling imbalance is related to the development of diseases such as abnormal angiogenesis, arterial hypertension, endothelial dysfunction and atherosclerosis (Jozefczuk et al., 2020). Another study identified 72 differential metabolites in thyroid carcinoma, and determined 5 metabolites with AUC values > 0.8, which could serve as potential signatures of thyroid carcinoma (Feng et al., 2019). Taken together, our results implied that the identified differential metabolites, particularly 1-methyladenosine, 9,10-DHOME, acetylcholine chloride, dodecanedioic acid, GMP, and N6-acetyl-L-lysine, may be candidate biomarkers for CHD, as well as mTOR signaling pathway, sphingolipid signaling pathway, FoxO signaling pathway, TCA cycle, AMPK signaling pathway, HIF-1 signaling pathway and cGMP-PKG signaling pathway may play crucial roles in CHD occurrence and progression. However, the specific effects of the differential metabolites and these involved pathways on CHD should be further explored.

Further, we excavated the correlation between gut microbiota and differential metabolites in CHD. Feng et al. (2019) combined 16s rRNA gene sequencing and metabolomics, and found that *Klebsiella* and *Coprococcus_3* were correlated with lipid-related metabolites; and *Lactobacillus*, *Megamonas*, and *Blautia* were related to benzenoid, amino acids, and flavonoids-related metabolites. Correlation analysis between gut microbiota and metabolites of another study showed that in polycystic ovary syndrome patients, serum testosterone level and estradiol level were, respectively, negatively correlated with *Prevotella_9* and *Clostridium* abundance; and luteinizing hormone level was positively correlated with *Bifidobacterium* abundance (Zhou et al., 2020). In this study, *Sphingomonae* had synergetic effects with vanylglycol, and 1-Methyladenosine; while had antagonistic effects with 2-dehydro-3-deoxy-D-xylonate and aminoadipic acid. *Pseudomonas* had synergetic effects with vanylglycol, while *Enterobacter* had antagonistic effects with trehalose. Five bacteria including *Nesterenkonia*, *Aliihoeflea*, *Granulicatella*, *Necropsobacter*, and *Aggregatibacter* had antagonistic effects with N,N-Dimethylhistidine. Additionally, the AUC of the conjoint analysis (0.908) was higher than that of gut microbiota species (0.742). All these findings indicated that the predictive effect of combined analysis may be better for CHD, and gut microbiota may participate in the physiological and pathological processes of CHD by regulating metabolites.

Although our study revealed characteristic changes in gut microbiota and serum metabolites in patients with coronary heart disease (CHD) through multi-omics analysis and identified potential biomarkers, we acknowledge that there are still some limitations. First, the sample size was relatively small (20 CHD patients and 20 healthy controls), which may limit the generalizability and statistical power of our findings. We plan to expand the sample size in future studies to validate these results. Second, all participants were elderly Chinese individuals, and we recognize that our findings may not be directly generalizable to other ethnic groups, age groups, or geographic regions. Third, although we controlled the diet during the sample collection period, we were unable to assess the long-term dietary habits of participants prior to hospitalization, which may have a significant impact on gut microbiota and metabolites. Fourth, we note that the absence of fecal metabolomics limits our ability to directly interrogate microbial-metabolite crosstalk at the gut interface. While serum metabolites provide valuable insights into systemic mechanisms and clinical translation potential, we understand that fecal metabolites are crucial for understanding localized gut interactions and microbial contributions to CHD pathogenesis. Finally, our study employed a cross-sectional design, reflecting the state of microbiota and metabolites at a single time point, and thus cannot establish causality. We believe that future studies should adopt a longitudinal design to evaluate the dynamic changes in gut microbiota and metabolites during the development and progression of CHD. We acknowledge these limitations and plan to address them in future research to enhance the robustness and generalizability of our findings.

## 5 Conclusion

In conclusion, untargeted metabolomics and 16s rRNA gene sequencing revealed characteristic changes in blood metabolites and gut microbiota in CHD. In CHD patients, the intestinal flora disorder was found, characterized by a decrease of beneficial bacteria (*Roseburia*, *Corprococcus*, and *Bifidobacterium*) and an increase the of pathogenic microbes (*Sphingomonas*, *Prevotella*, *Streptococcus*, *Desulfovibrio*, and *Shigella*) in gut. *Sphingomonas* may be closely related to CHD, and may serve as a crucial biomarker of intestinal flora for CHD. Additionally, a total of 155 differential metabolites, including 1-methyladenosine, 9,10-DHOME, acetylcholine chloride, dodecanedioic acid, GMP, and N6-acetyl-L-lysine, may serve as candidate biomarkers for CHD occurrence and progression. This work identifies the key functional gut microbiota and potential biomarkers associated with the pathogenesis of CHD; and provides novel promising targets for the diagnosis and therapy of CHD.

## Data Availability

Original contributions presented in the study are included in the article and supplementary material. The gut microbiota (16S rRNA) and blood metabolomics (LC-MS) data associated with this study are available in the Figshare repository, doi: 10.6084/m9.figshare.28750520.

## Footnotes

^1^ http://qiime.org/

^2^ http://greengenes.secondgenome.com/

## Supplementary material

Supplementary Figure 1 Quality control and quality assessment of all the samples using Principal Component Analysis.

Supplementary Figure 2 The clustering heatmap of the identified differential metabolites based on the thresholds of VIP ≥ 1 and *p*-value ≤ 0.05.

Supplementary Figure 3 Receiver operating characteristic (ROC) and area under curve (AUC) of some related differential metabolites.
