# Supplementary figures and images for: Multi-omics analysis identifies Sphingomonas and specific metabolites as key biomarkers in elderly Chinese patients with coronary heart disease
Source: Front Microbiol. 2025 Apr 23;16:1452136. doi: 10.3389/fmicb.2025.1452136 (PMC12058083; doi:10.3389/fmicb.2025.1452136)

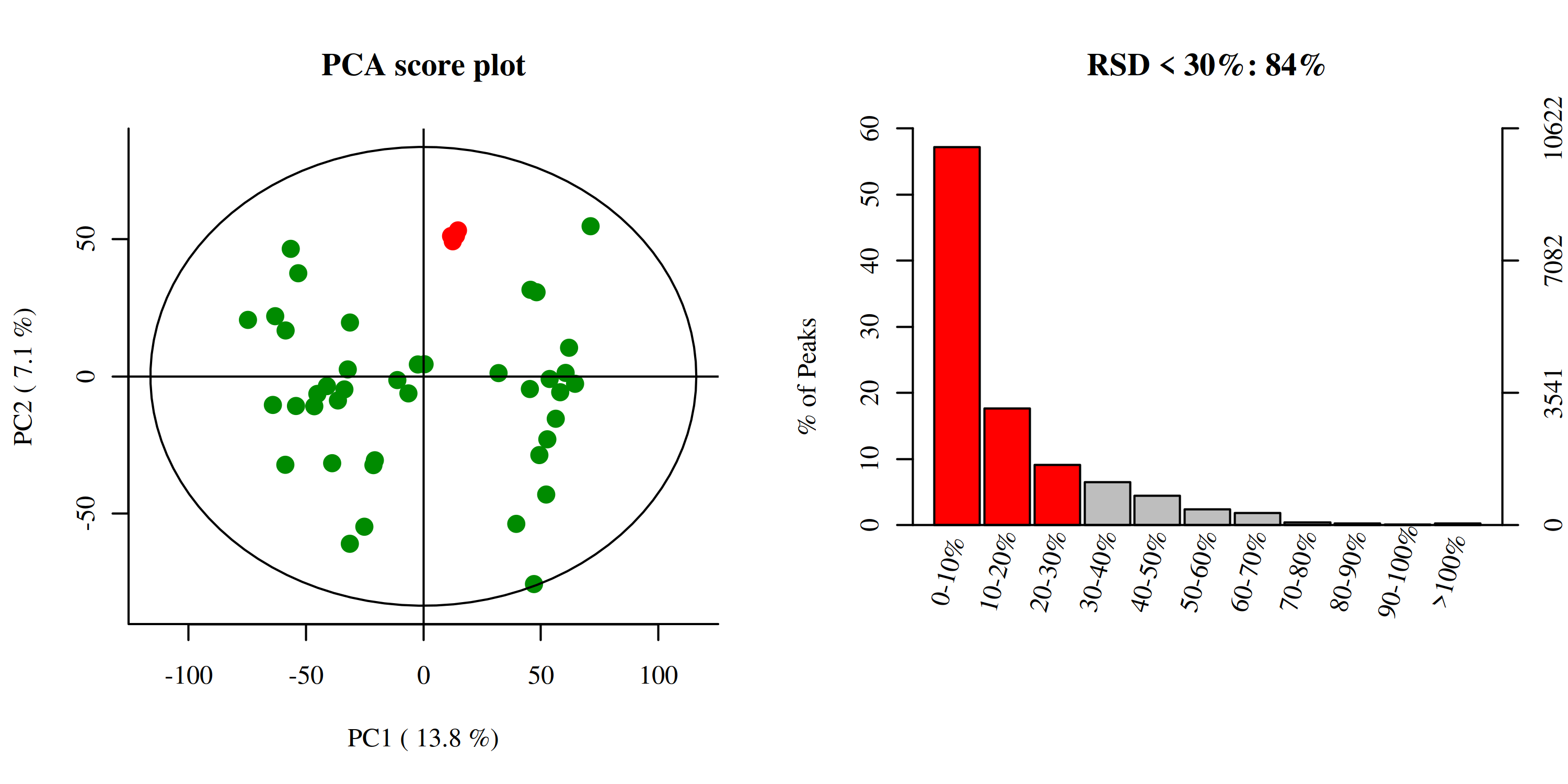

Supplement: Supplementary Figure 1 — Quality control and quality assessment of all the samples using Principal Component Analysis. [file Image_1.PNG]

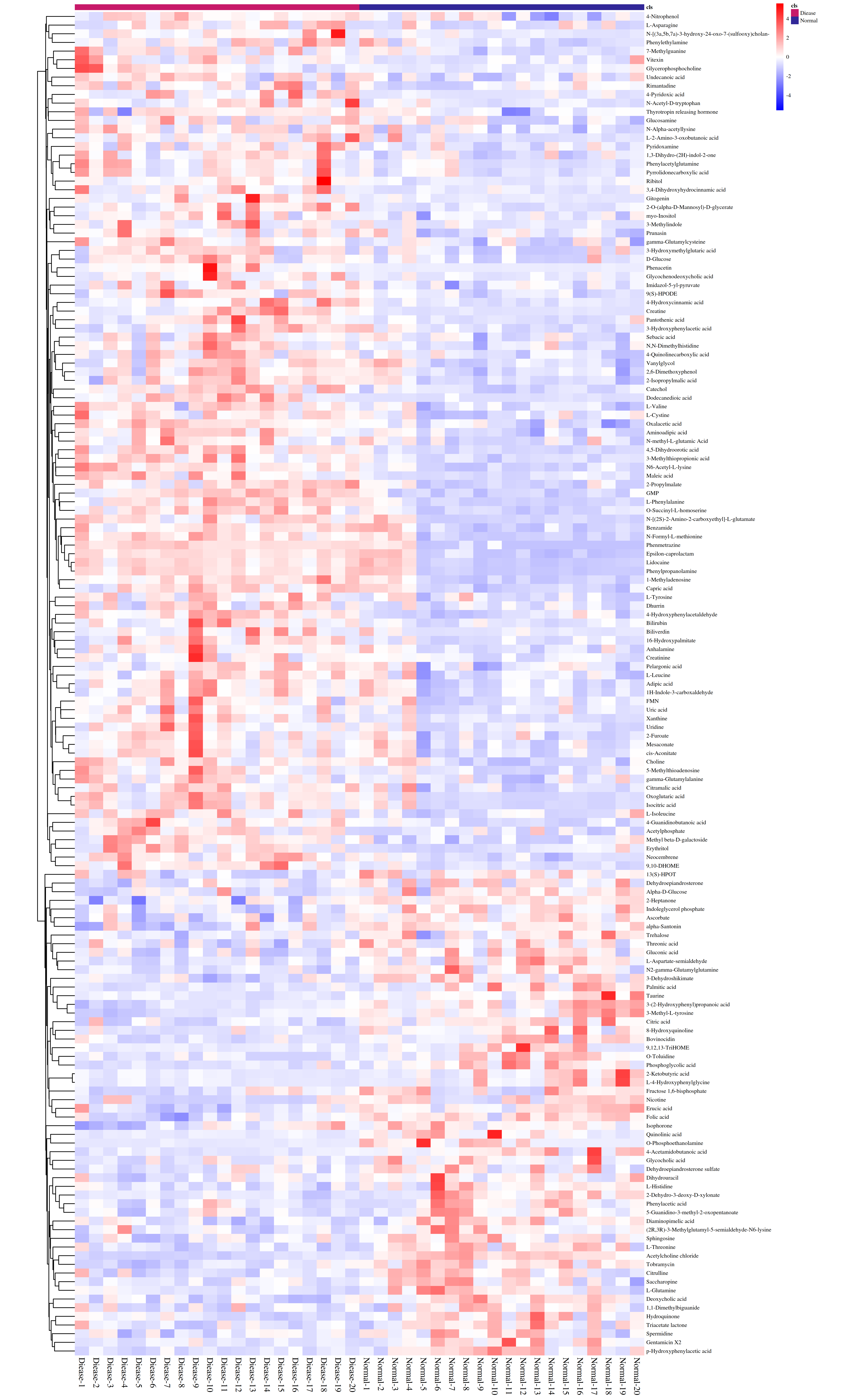

Supplement: Supplementary Figure 2 — The clustering heatmap of the identified differential metabolites based on the thresholds of VIP ≥ 1 and p-value ≤ 0.05. [file Image_2.PNG]

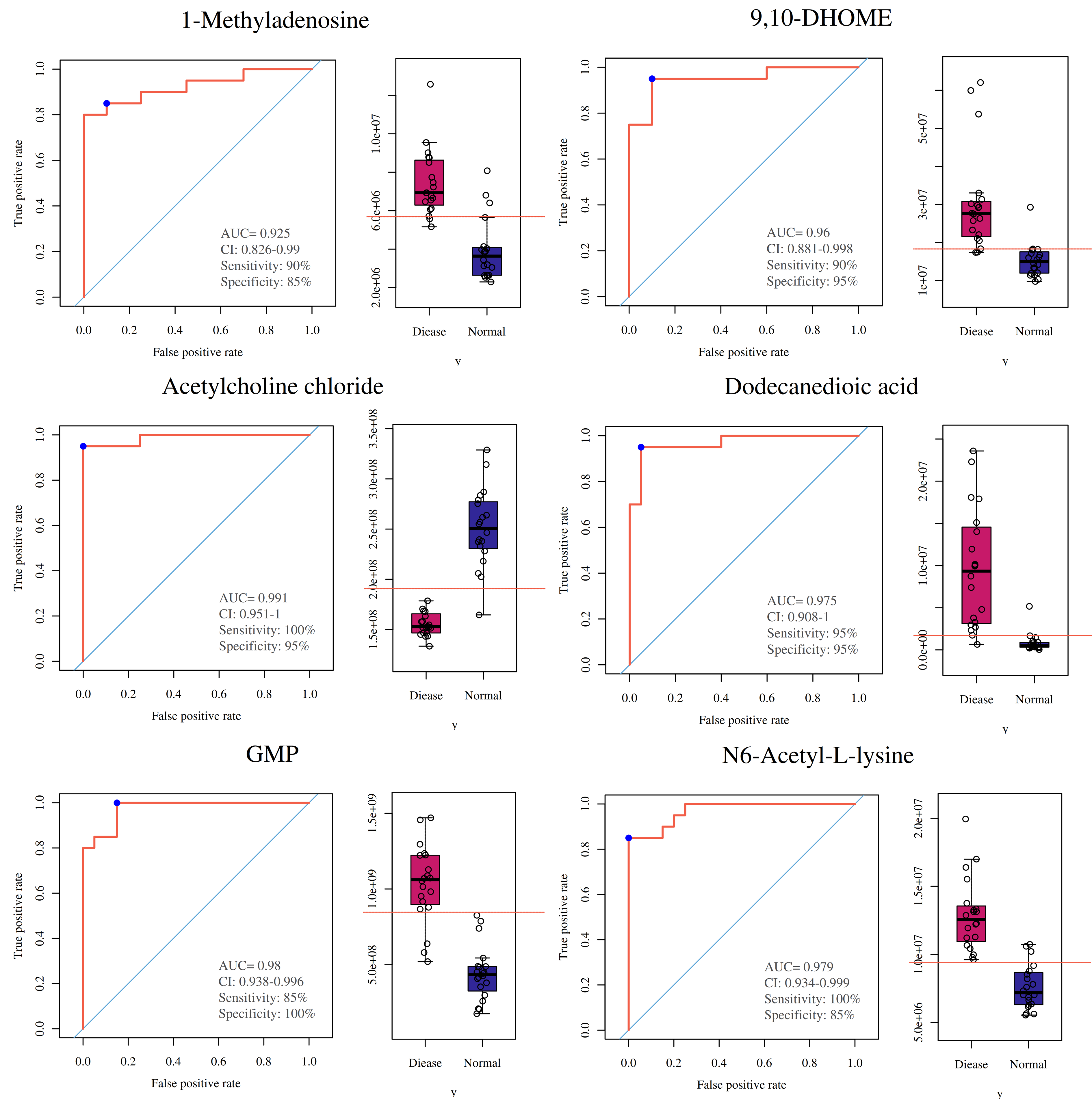

Supplement: Supplementary Figure 3 — Receiver operating characteristic (ROC) and area under curve (AUC) of some related differential metabolites. [file Image_3.PNG]
